# Novel Use of a Closed Liposuction System: Treatment of an Acute Morel-Lavallée Lesion of the Upper Extremity

**DOI:** 10.7759/cureus.8437

**Published:** 2020-06-04

**Authors:** Anooj Patel, Corinne Wee, Mitchell K Ng, Anand Kumar, Donald Harvey

**Affiliations:** 1 Plastic Surgery, Case Western Reserve University School of Medicine, Cleveland, USA; 2 Plastic Surgery, University Hospitals Cleveland Medical Center, Cleveland, USA; 3 Orthopaedic Surgery, Maimonides Medical Center, Brooklyn, USA; 4 Plastic Surgery, University Hospitals, Cleveland, USA

**Keywords:** morel-lavallée lesions, soft tissue surgery, suction-assisted lipectomy

## Abstract

Morel-Lavallée lesions (MLL) are closed post-traumatic soft tissue shear injuries that occur between fascial planes and may result in tissue loss. Current treatment options for MLL include percutaneous drainage and open irrigation and debridement. A few cases of suction-assisted lipectomy (SAL) have been described for subacute and chronic MLL of the lower extremity. We present the first case report of using a closed SAL system to treat an acute MLL of the upper extremity. A 78-year-old female with right forearm MLL presented after blunt force trauma while on apixaban. After inpatient monitoring and anticoagulant drug clearance, a closed system SAL was performed to evacuate the hematoma and prevent ischemia of overlying soft tissues. Treatment outcomes were measured by clinical exam and CT imaging. Pre-operative diagnostic CT scan demonstrated a 4.8 x 6.6 x 13 cm fluid collection between fascial layers of the right forearm. SAL resulted in the evacuation of 300 cc of coagulated blood. Post-operative CT imaging of the right upper extremity did not show any measurable fluid collection. Clinical exam demonstrated resolution of swelling and soft tissue compromise. The patient reported significant pain reduction, resumed her anticoagulation, and was discharged home. There were no notable complications at her three month post-operative visit. Consideration of a constant low-pressure SAL system can successfully treat MLL in the acute period. This system is relatively minimally invasive, results in faster healing times compared to open debridement, and still results in effective hematoma evacuation.

## Introduction

Morel-Lavallée lesions (MLL) are traumatic closed soft tissue injuries that occur as a result of shearing forces that separate superficial and deep fascial layers. They are commonly seen in the greater trochanteric region, flank, knee, and buttocks [1]. There are limited reports of this lesion in the upper extremity and even fewer in the acute setting [2,3].

Clinical features suspicious for MLL include a soft and fluctuant area of skin with possible ecchymosis, contour deformities, skin hypermobility, decreased sensation, or a palpable bulge to an area that has been subjected to tangential forces [1]. These lesions can occur acutely or develop days after the injury depending on the mechanism and location [3].

Urgent diagnosis and management is indicated to remove fat, blood clots, and lymph that may serve as a medium for bacterial colonization [3]. Diagnosis is based on clinical history, physical exam, and imaging. MRI is often preferred as it can identify the extent of the lesion as well as any underlying bone fractures. Chronicity of lesions may be determined from signs of capsule formation and varying intensities on T1- and T2-weighted images [4]. Ultrasound has also been used as a diagnostic method; a case report by LaTulip et al highlights its utility in the emergency room where it is both cost-effective and accurate [5].

Current treatment for MLL depends on the size, location, and severity. It includes close observation without surgical intervention, compression banding, percutaneous drainage, serial aspirations, and open debridement and irrigation [1,3,4]. Novel treatments, such as doxycycline-induced sclerodesis, have been shown to be successful in persistent lesions [6]. A few cases of suction-assisted lipectomy (SAL) have also been described in the literature, but only in the case of chronic lesions of the lower extremity [7].

We present the first case report of using a closed SAL system to treat an acute MLL of the upper extremity.

## Case presentation

A 78-year-old female with right forearm MLL presented after blunt force trauma while on apixaban. She was initially admitted due to concern for compartment syndrome due to significant swelling. She was evaluated by plastic surgery, which recommended holding apixaban, elevating the extremity, and serial physical examinations. Once serial exams were not concerning for evolving hematoma, therapeutic lovenox was started on hospital day 3. She was taken to the operating room after apixaban clearance due to concern for developing skin necrosis. Two incisions were made using a 15-blade scalpel, one proximally and one distally, over the most prominent areas of hematoma. SAL was used to remove approximately 250 cubic centimeters (cc) of coagulated blood, and additional 50 cc was removed with manual pressure. A closed system provides a constant 300 millimeters mercury (mmHg) vacuum pressure compared to commonly higher and variable negative pressures with standard liposuction. These incisions were left open to allow for additional drainage, and a compression dressing consisting of Xeroform (Curad Medline Industries, Mundelein, IL), abdominal pads (ABDs; Covidien, Mansfield, MA), Kerlix (Covidien, Mansfield, MA), and ace bandages were applied over the area. Treatment outcomes were measured by clinical exam, patient-reported symptoms, and CT imaging. 

**Figure 1:**
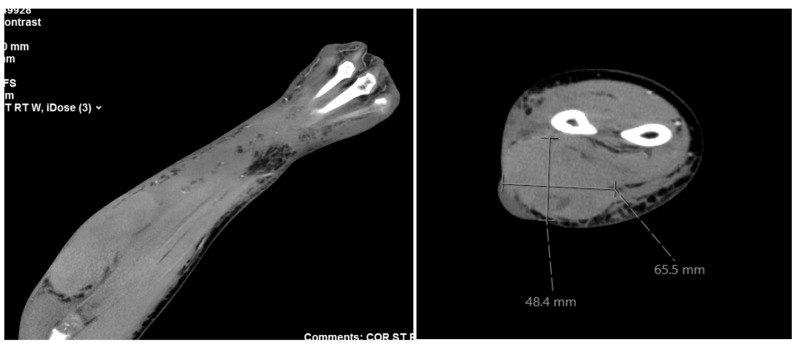
Pre-operative CT demonstrating a 4.8 x 6.6 x 13 cm fluid collection between fascial layers of the right forearm

Pre-operative diagnostic CT scan demonstrated a 4.8 x 6.6 x 13 cm fluid collection between fascial layers of the right forearm (Figure 1).

**Figure 2:**
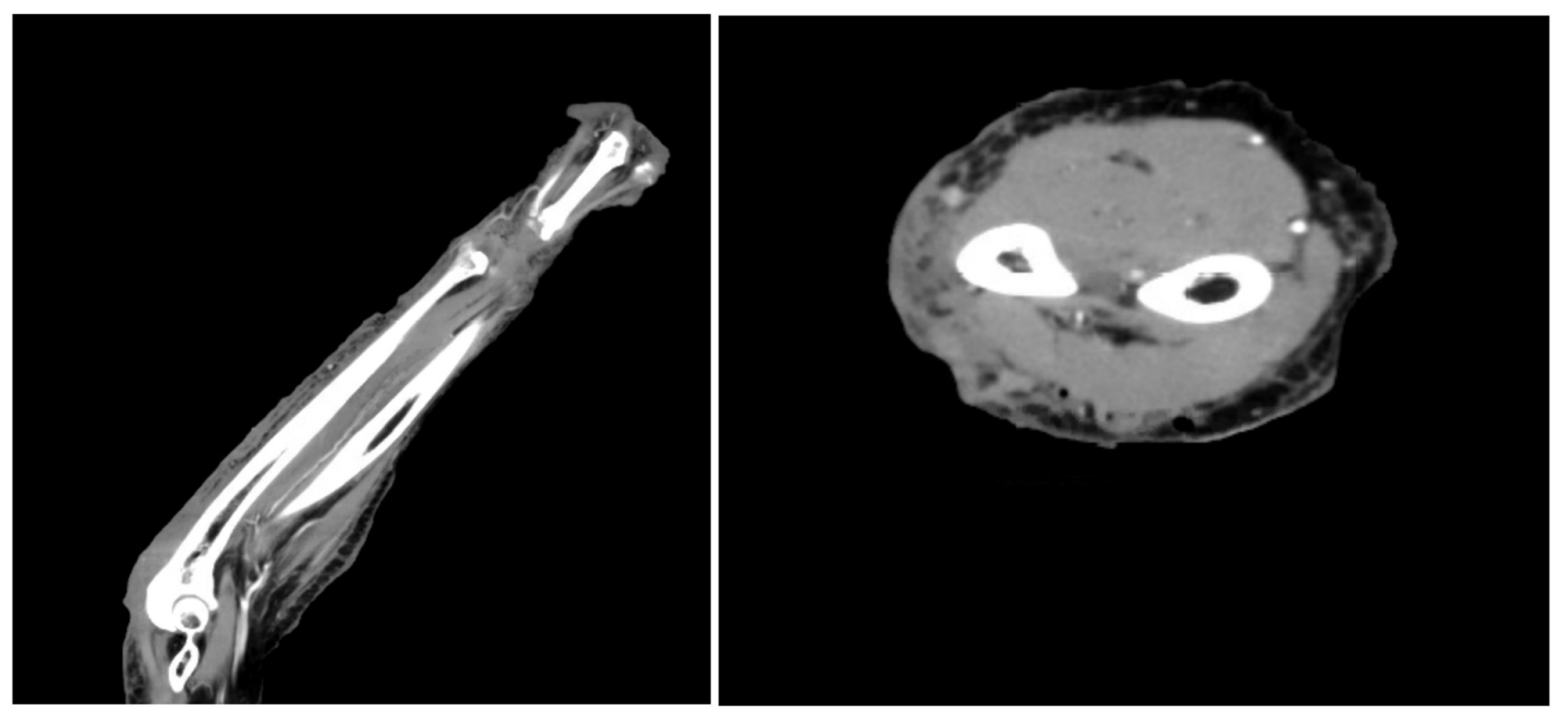
Post-operative CT scans showing no measurable fluid collection

SAL resulted in the evacuation of 300 cc of coagulated blood. Post-operative CT imaging did not show any measurable fluid collection (Figure 2).

Her pressure dressing was removed on post-operative day 1, and clinical exam demonstrated resolution of swelling and soft tissue injury (Table 1).

**Table 1:** Course of patient symptoms (pre-operative, immediately post-operative, and at 30 days post-operative)

	Pre-op	Post-op	30-day visit
Symptoms	Pain, swelling	Post-operative pain, pressure pain improved	Absent
Pain	Yes	Greatly improved	Absent
Upper extremity exam	Significant swelling, neurovascularly intact	Swelling resolved, neurovascularly intact	Swelling, ecchymosis resolved, skin healed, neurovascularly intact
Skin findings	Small ulceration, ecchymosis	Residual ecchymosis	Well healed

**Figure 3:**
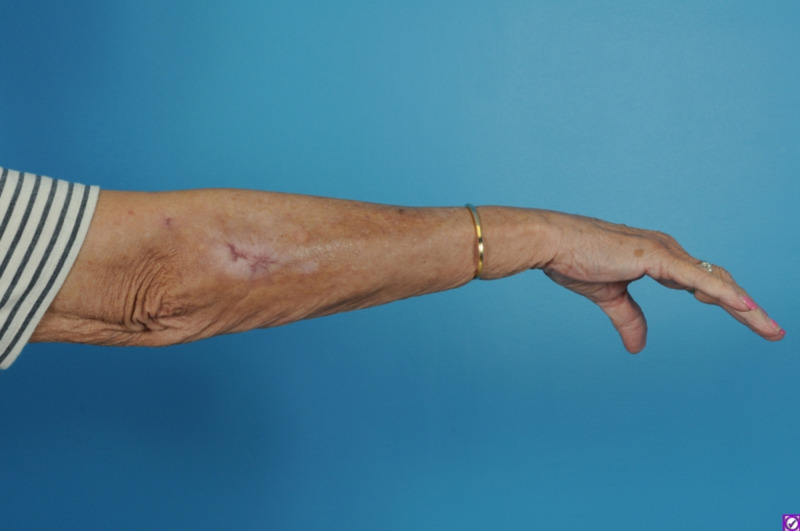
Three-month post-operative photo

The patient reported significant pain reduction, resumed her anticoagulation on post-operative day 1, and was discharged home on post-operative day 2. There were no notable complications at her three-month post-operative visit; all incisions were well healed (Figure 3).

## Discussion

Current treatment options for MLL are not well studied and vary significantly based on location and size of the lesion. Given the anatomic complexity of the upper extremity, safe and effective treatment options for MLL in this area are important. This case report hopes to address the lack of published literature on upper extremity MLL.

Ineffectively treated lesions may result in significant complications, including bacterial colonization, compartment syndrome, and tissue necrosis. While conservative treatment, percutaneous drainage, and open debridement are common treatment options for lesions, they each have limitations and side effects. Furthermore, these treatment options are poorly studied in the upper extremity and are mainly described for the lower extremity. Conservative treatment mainly involves observation, compression banding, and sclerodesis and is reserved for smaller lesions. Similarly, percutaneous drainage often involves serial aspirations of smaller lesions and is often unable to fully evacuate the lesion. Limitations of these options include lesion recurrence, repeated office visits, lesion chronicity, pseudocyst formation, and bacterial infection as a result of suboptimal evacuation [3,4].

Larger lesions that cannot be percutaneously drained often require open debridement and irrigation. This approach may compromise the tissue’s subdermal vascular supply, sometimes leading to poor or delayed wound healing and soft tissue necrosis [3]. Furthermore, this surgical approach can lead to contour deformities, scar formation, and poor cosmesis.

SAL is an option that preserves the tissue subdermal plexus with only a small slit incision where the suction device enters. This minimizes both scar and wound healing complications when compared to an open approach. SAL allows for evacuation of the entire fluid collection in contrast to percutaneous drainage, and therefore may be beneficial in larger lesions. Additionally, in patients with a history of anticoagulant use, SAL may provide an additional benefit to open procedures by minimizing blood loss.

A disadvantage of SAL is that any dead space cannot be directly closed as in an open incision. This may increase the risk of infection and recurrence in certain anatomic locations. However, one option would be to thread a drain through one of the small incisions to allow for continued drainage and elimination of dead space.

The distinguishing features of our case report include low-pressure liposuction treatment in the acute setting, with the goal of preventing tissue ischemia, while mitigating symptoms rather than to release any capsule, pseudocyst, or scar tissue. Additionally, our case focuses on the upper extremity, a relatively rare and more sensitive location for such a lesion [2,3]. Previous case reports have described SAL as a treatment option of chronic and lower extremity MLL. A case report, in which a patient suffered a 15 x 15 cm MLL of the lateral right thigh two years prior, received liposuction treatment. The goal in that case report was to fix overlying contour deformity due to scar contracture. This differs from the goal in our case report, which was to prevent tissue ischemia due to pressure from hematoma formation [7]. Another lower extremity case report utilized liposuction to treat a subacute (three weeks prior) MLL of the left lateral thigh with capsule formation [8]. Limited upper extremity case reports exist for chronic lesions with pseudocyst formation and in pediatric patients [9].

Further study is needed to understand the long-term outcomes and indications of SAL in comparison to open debridement and irrigation. These studies should investigate rates of infection, recurrence, contour irregularities, and skin necrosis. Further investigation is also needed to compare different SAL systems and understand the optimal SAL pressures to prevent damage of soft tissue and surrounding structures in different anatomic locations.

## Conclusions

MLL is a post-traumatic soft tissue degloving injury that may result in serious complications. There is a lack of literature on the management options for these lesions in the upper extremity. In the acute setting, prompt diagnosis and management of these lesions is imperative. Low-pressure SAL was an effective treatment option for acute MLL of the upper extremity in an elderly patient with a history of anticoagulant use. The constant low-pressure SAL system can prevent damage to the tissue’s underlying vascular supply, while preventing contour deformities or scar formation. SAL allows for the treatment of larger lesions in comparison to percutaneous drainage and faster healing time when compared to open debridement.
